# Discriminant Analysis of Essay, Mathematics/Science Type of Essay, College Scholastic Ability Test, and Grade Point Average as Predictors of Acceptance to a Pre-med Course at a Korean Medical School

**DOI:** 10.3352/jeehp.2008.5.4

**Published:** 2008-12-22

**Authors:** Geum-Hee Jeong

**Affiliations:** Division of Nursing, Institute of Medical Education and Office of Admissions Affairs, Hallym University, Chuncheon, Korea.

**Keywords:** College Scholastic Ability Test, Entrance Examination, Essay, General Point Average, Mathematics/Science, Medical School

## Abstract

A discriminant analysis was conducted to investigate how an essay, a mathematics/science type of essay, a college scholastic ability test, and grade point average affect acceptance to a pre-med course at a Korean medical school. Subjects included 122 and 385 applicants for, respectively, early and regular admission to a medical school in Korea. The early admission examination was conducted in October 2007, and the regular admission examination was conducted in January 2008. The analysis of early admission data revealed significant F values for the mathematics/science type of essay (51.64; *P*<0.0001) and for grade point average (10.66; *P*=0.0014). The analysis of regular admission data revealed the following F values: 28.81 (*P*<0.0001) for grade point average, 27.47 (*P*<0.0001) for college scholastic ability test, 10.67 (*P*=0.0012) for the essay, and 216.74 (*P*<0.0001) for the mathematics/science type of essay. Since the mathematics/science type of essay had a strong effect on acceptance, an emphasis on this requirement and exclusion of other kinds of essays would be effective in subsequent entrance examinations for this premed course.

## INTRODUCTION

In 2007, the Korean Ministry of Education prohibited disclosure of students' percentile scores or total scores on the College Scholastic Ability Test (CSAT), which was used as a university entrance requirement. Students and universities are now only provided with the nine scale scores. The government's goal is to diminish the impact of the CSAT when recruiting students, and it has encouraged universities to develop various other tools for recruiting students. However, admission officers have found it difficult to choose among first-year applicants to pre-med courses at Korean medical schools because many applicants have earned the highest possible grades in subjects such as Korean language, English, mathematics, and science. In addition, grade point average (GPA) is not a helpful discriminating variable because high schools vary greatly in quality levels. Therefore, some admission officers have decided to use essays and mathematics/science types of essays when recruiting first-year students to pre-med courses. In this test case, professors volunteered to create and administer these tests for early admission in October 2007 and regular admission in January 2007. After testing, a discriminant analysis was conducted to determine the discriminating power of each variable on first-year recruitment: an essay, a mathematics/science type of essay, CSAT scores, and GPA. The results should help admission officers to assess students more efficiently during the next entrance examination.

## MATERIALS AND METHODS

A total of 469 students applied for early admission in November 2007; of these, 347 did not meet the minimum CSAT scores. The remaining 122 applicants were subjected to more testing; of these, 91 failed, although they had met the minimum CSAT requirements. The remaining 31 applicants passed, either immediately or after additional attempts. Five applicants withdrew from consideration, although they had met the requirements. The minimum CSAT criterion for early admission was six or fewer grades in four subjects such as Korean, English, mathematics, and science. A discriminant analysis using DBSTAT [1] was conducted to assess three other variables: as essay, a mathematics/science type of essay, and GPA.

A total of 385 students applied for regular admission and completed the essay and mathematics/science type of essay. Of these, a total of 70 applicants passed, including 17 who later withdrew from consideration. Overall, 79 students were admitted during early and regular admission. Similar discriminant analyses were conducted on all four variables: the essay, the mathematics/science type of essay, GPA, and CSAT. Correlations among the four variables and comparisons according to sex were also investigated for both admission times.

## RESULTS

Analysis of early admission data revealed no significant correlation between GPA and the essay and no correlation between the essay and the mathematics/science type of essay. Variables that affected acceptance did not include the essay (F=3.13, *P*=0.0559) but did include the mathematics/science type of essay (F=51.64, *P*<0.0001) and GPA (F=10.66, *P*=0.0014). Among successful applicants, females had higher GPAs than males (*P*=0.0120).

The analysis of the regular admission data revealed a correlation among the four variables (*P*<0.05). Correlation coefficients were as follows: 0.7503 between GPA and CSAT, 0.1120 between GPA and the essay, 0.3486 between GPA and the mathematics/science type of essay, and 0.1261 between the essay and the mathematics/science type of essay. Discriminant analyses showed that all F values were significant: 28.81 for GPA; 27.47 for CSAT; 10.67 for the essay; and 216.74 for the mathematics/science type of essay. No significant differences according to sex were observed for any of the four variables within the total group of applicants. Of the successful applicants, females had significantly higher GPA scores than males (*P*=0.0261), but no significant differences were observed with regard to the essay and the mathematics/science type of essay according to sex.

Among those applying for early admission, scores for the mathematics/science type of essay ranged from 6.3-55.7 and scores for the essays ranged from 6.7-25.0. Among those applying for regular admission, scores for the mathematics/science type of essay ranged from 2.8-58.0 and scores for the essays ranged from 10.0-27.3. The maximum score for the mathematics/science type of essay was 65, and the maximum score for the essay was 35.

## DISCUSSION

These results verify that the mathematics/science type of essay was the most powerful predictor of admission among the several variables used during the entrance examination to the pre-med course. The large range in scores for the mathematics/science type of essay also indicated that it effectively discriminated between successful and unsuccessful applicants; much less variation appeared among scores for the essays, GPAs, and CSAT. A high F value does not always signify great discriminant power; the *P*-value is more important to this interpretation. In this study, the F value for the mathematics/science type of essay was exceptionally large and the *P*-value was very low; therefore, the mathematics/science type of essay can be considered the most powerful discriminant.

This study was limited in its design. Passing or failing the entrance examination depended on scores obtained on the variables used in this study, so confounding is likely when applying each variable portion to the total score. In addition, the high correlation between GPA and CSAT among those applying for regular admission may have been an additional confounding factor. Still, the data reveal a tendency toward the predictive power of the mathematics/science type of essay in regard to successful application for admission.

If the essay-type test is to be repeated during the next entrance examination to the pre-med course, it would be more efficient to administer only a mathematics/science type of essay rather than another kind of essay, as the latter involves unduly laborious work for professors.
